# Exploitation of Complex Abelian Point Groups in Quantum-Chemical Calculations

**DOI:** 10.1021/acs.jpca.6c00998

**Published:** 2026-06-09

**Authors:** Marios-Petros Kitsaras, Stella Stopkowicz

**Affiliations:** † Laboratoire de Chimie et Physique Quantiques (UMR 5626), 246574 Université de Toulouse, CNRS, Bat. 3R1b4, 118 route de Narbonne, 31062 Toulouse, Cedex 09, France; ‡ Physikalische und Theoretische Chemie, 9379 Universität des Saarlandes, Campus, Geb. B2.2, 66123 Saarbrücken, Germany; § Department Chemie, Johannes Gutenberg-Universität Mainz, Duesbergweg 10-14, 55128 Mainz, Germany; ∥ Hylleraas Centre for Quantum Molecular Sciences, Department of Chemistry, University of Oslo, P.O. Box 1033 Blindern, 0315 Oslo, Norway

## Abstract

Quantum-chemical calculations often make use of point-group theory to exploit molecular symmetry, resulting in a reduction of the computational cost and in insights into the electronic structure. This exploitation is often limited to subgroups of *D*_2*h*_ that are Abelian with real characters. Here, we extend the symmetry exploitation to Abelian point groups with complex characters. Such point groups are often encountered in calculations that involve finite magnetic fields, though their occurrence is not limited to these cases alone. We present the evaluation of integrals over symmetry-adapted orbitals using the double-coset decomposition, as well as the use of these symmetries in the contractions needed within post-Hartree-Fock calculations in the context of block tensors. Efficiency gains are discussed for four simple hydrocarbons that exhibit a complex Abelian point group in the presence of a magnetic field.

## Graphical abstract

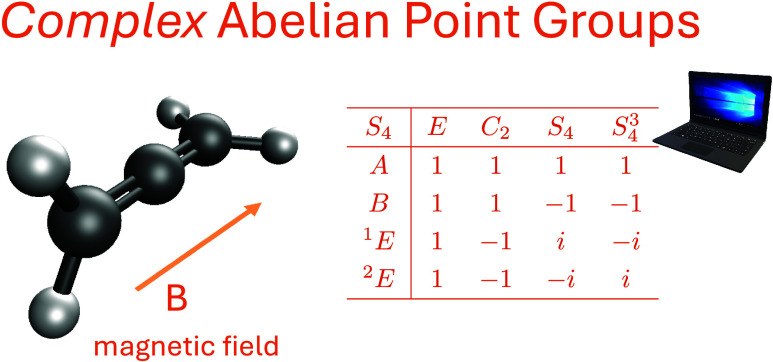

## I Introduction

Symmetry is a fundamental organizing principle in chemistry because it rigorously constrains how molecular structures and electronic states transform under geometric operations. It provides a powerful framework to interpret molecular properties, such as the classification of vibrational modes in spectroscopy, the assignment of molecular orbitals, and the prediction of allowed electronic transitions. As an example, standard textbooks such as Chemical Applications of Group Theory (Cotton, Wiley)^1^ illustrate how group theory links molecular structure, observables, and physical behavior.

In quantum-chemical computations, symmetry is exploited both to interpret electronic structure and to reduce computational cost in terms of both memory requirements as well as computation time.^2–13^ The central principle is that exact solutions to the Schrödinger equation transform like one of the irreducible representations (IRREP) of the molecular point group (PG). For approximate solution this is not guaranteed.^14^ However, while symmetry-broken solutions can sometimes be advantageous, enforcing solutions that transform as the IRREPs of the molecular PG is preferred in most cases. In this way, due to resulting selection rules, the computation and handling of integrals, amplitudes, and intermediate quantities that vanish due to symmetry can be avoided. Most often such symmetry implementations are based on Abelian PGs with real-valued characters as wave functions of interest are formulated in a purely real manner in the majority of quantum-chemical calculations. However, there are situations in which real representations are insufficient, motivating the use of Abelian PGs with complex-valued characters to fully exploit molecular symmetry and reduce computational cost. This is true in particular in cases where the wave function of the system under consideration becomes complex. Complex wave functions occur, for example, for molecules in finite magnetic fields^15^ or in relativistic calculations that consider spin–orbit effects in a nonperturbative manner.^16^ But even in standard field-free nonrelativistic calculations, complex wave functions can arise. This includes, for example, open-shell Π, Δ, etc. states as eigenvalues of the angular momentum operator, as well as non-Hermitian parametrizations of the Hamiltonian, like in equation-of-motion coupled cluster (EOM-CC) theory, when treating accidentally degenerate excited states of molecules whose symmetry is described by a complex Abelian PG (e.g., excited states of B­(OH)_3_, *C*_3*h*_).^17,18^

In general, symmetry exploitation in quantum-chemical programs occurs throughout the calculations, starting from the level of integral evaluation up to the tensor-contraction level involved in electronic-structure approximations (self-consistent field (SCF), post-Hartree–Fock (HF), etc.). At the level of integral evaluation, symmetry can be used to reduce the number of integrals that need to be computed. Two widely used approaches are the petite-list method^3,4^ and the double-coset decomposition (DCD).^2^ Both function by identifying the symmetry-equivalent combinations of basis functions and avoid repeated evaluations of symmetry-equivalent integrals. While the petite-list approach is formally and algorithmically simpler without any drawback in the case of non-Abelian groups, the resulting integral matrices are not symmetry-adapted and the rule of vanishing integrals cannot be directly exploited. The DCD on the other hand leads to a block structure of the integral matrices as it implicitly symmetry adapts the basis functions. While a formulation of the DCD in the case of non-Abelian PGs has been derived, it relies on the full matrices of the IRREPs rather than their characters, which makes its practical implementation more involved. For HF and post-HF electron correlation treatments, all quantities are typically expressed in terms of symmetry-adapted molecular orbitals (MOs). In this context, the direct-product decomposition (DPD) scheme^7^ enables the systematic symmetry blocking of amplitudes and intermediates, allowing efficient exploitation of Abelian PGs. Moreover, recent work has extended symmetry exploitation using the DPD scheme to non-Abelian PGs, demonstrating potential computational savings in many-body methods when such symmetries are fully utilized.^19^

In this work, we focus on the case of molecules in a finite magnetic field. Symmetry is typically reduced due to the anisotropy of the magnetic field and the rotational degrees of freedom of the molecule are not invariant. Nonetheless, molecules are in many cases oriented in a symmetric manner with respect to the magnetic field and some symmetry elements may survive,^20,21^ motivating the exploitation of symmetry in finite-magnetic field calculations. As such, investigating the optimized structure for symmetric orientation of the magnetic field is crucial as they are often saddle points (minima or maxima) of the potential energy surface.^22^ Symmetry handling for Abelian PGs with real characters in the cfour program^23,24^ can be traced back to the corresponding implementation in the aces ii^25,26^ program package. In the more recent mint^27^ integral package within cfour, symmetry exploitation was implemented by Gauss for the evaluation of integrals over both standard Gaussian basis functions and London orbitals.^28^ In the context of finite magnetic fields, this symmetry handling was further extended to the HF-SCF procedure itself. For subsequent post-HF calculations, a corresponding symmetry handling was introduced within the qcumbre^29,30^ program.^22^ These developments have enabled the symmetry exploitation at the finite-field (ff) HF, second-order Møller–Plesset perturbation theory, and various flavors of CC and EOM CC levels of theory.^17,22,29,31–36^ Here, we extend this symmetry handling to account also for Abelian PGs with complex characters as such PGs often describe the molecular symmetry in the presence of a magnetic field.^10^ We note in this context that, recently, tools for symbolic symmetry analysis specialized for magnetic PGs have been developed.^12,37^

We present an implementation of Abelian PGs with complex characters in a quantum-chemical framework. We first revisit the DCD for such groups and derive working equations suitable for a practical integral evaluation. Building on this, we present the theoretical background for the respective exploitation of symmetry in HF and post-HF correlation methods, including CC, EOM-CC, and the evaluation of molecular properties. Lastly, we report on the resulting computational savings and performance improvements, illustrated by ground state CCSD energies.

## II Theoretical Methods

Complex Abelian PGs possess all the properties of real Abelian PGs that make them attractive for their exploitation within quantum-chemical calculations. They only have one-dimensional IRREPs, and their symmetry elements commute. However, they have complex entries in their character tables and their matrix respresentations,^38^ which hinders their exploitation, as many quantum-chemical program packages are restricted to real numbers. IRREPs of a group with complex entries come in complex-conjugate pairs. This practically means that for each IRREP with complex matrix representations, Γ^α^, there exist an IRREP Γ^α*^ whose matrices are the complex conjugate of each other without them being equivalent. The direct product between this complex-conjugate pair results in the totally symmetric IRREP

$$\Gamma^{\alpha }⊗\Gamma^{\alpha *}=\Gamma^{1}$$

In this work, the theoretical framework for the exploitation of complex Abelian PGs in quantum-chemical calculations is presented. It encompasses both the employment of these PGs in the calculation of integrals over atomic basis functions as well as the symmetry handling in SCF and post-HF approaches. Key differences and additional considerations with respect to the standard real Abelian PGs, i.e., *D*_2*h*_ and its subgroups, will be discussed.

### A Double-Coset Decomposition in Complex Abelian Point Groups

The DCD for the calculation of integrals of atomic orbitals has been first presented by Davidson^2^ and has later been extended to integral derivatives.^5,6^ Closely related developments in the exploitation of symmetry for integral evaluation were implemented earlier and reported by Almlöf.^26^ We refer the reader to ref 39 for a pedagogical introduction to the DCD. Here, we limit the presentation to the specific case of complex Abelian PGs. For clarity, the notation used in this manuscript for point-group theory is summarized in Table [Table tblI].

**1 tblI:** Overview of Notations

notation	use
*G*_*i*_	symmetry element
${Ĝ}_{i}$	symmetry operation
G	point group
Γ^α^	IRREP with index α
γ_*G*_*i*__^α^	character of IRREP α and symmetry element *G*_*i*_
*n*_*G*_	number of elements within group G
*R*	a specific choice of F and H subgroups for the construction of double cosets $FG_{i}H$
*R*_*k*_	double-coset representatives that generate all distinct double cosets for *R*
*n*_DCR_^*R*^	number of distinct double cosets for *R*
λ_*R*_	degeneracy of the double cosets generated for *R*
χ_μ,*A*_	function in the AO set with index μ and atomic center *A*
*X*_μ,*A*_^α^	function in the SAO set with index μ, delocalized over atomic centers of type *A*, transforms as IRREP Γ^α^
*G*_*i*_(*A*)	symmetry-equivalent center to *A*, into which *A* transforms after applying symmetry operation *Ĝ*_*i*_
[*A*,*B*]	a pair of centers *A* and *B*
U	stabilizer of center *A*
Γ_*p*_	IRREP of symmetry-adapted object *p*

In this work, the calculation of one- and two-electron integrals takes place in the AO basis {χ_μ,*A*_}. The index μ enumerates the AOs centered at nucleus *A*. However, since the AOs do not necessarily transform according to the IRREPs of the PG of the molecule, it is useful to work with symmetry-adapted atomic orbitals (SAOs) {*X*_μ,*A*_^α^}. SAOs are enumerated by index μ as well, and transform according to IRREP Γ^α^. Furthermore, they are typically delocalized over all symmetry-related sites obtained by applying the symmetry operations of the group to center *A*. Given a complex Abelian PG G with symmetry elements {*G*_*i*_}, a transformation from the AO to the SAO set can be defined as

$${\mathrm{P̂}}^{\alpha }\chi_{\mu ,A}=\frac{1}{n_{G}}\overset{n_{G}}{\underset{i}{\sum }}\gamma_{G_{i}}^{\alpha *}{Ĝ}_{i}\;\chi_{\mu ,A}=X_{\mu ,A}^{\alpha }$$ 1

using the projection operator ${\mathrm{P̂}}^{\alpha }$ . Here, γ_*G*_*i*__^α^ is the character of symmetry element *G*_*i*_ in the IRREP Γ^α^ and *Ĝ*_*i*_ is the operator associated with the corresponding symmetry element. Note that unlike in the case of real Abelian groups, the complex conjugation for γ_*G_i_*_^α^ cannot be omitted. A drawback of an explicit transformation such as that in eq 1 is that the summation over all *n*_*G*_ elements of the group typically leads to redundant contributions, i.e., the same functions are generated multiple times. Rather than proceeding in this manner and subsequently factorizing the terms in the final expression, the DCD is employed to eliminate these redundancies straight from the beginning, as explained below.

A double coset forms a subset of the elements of the group without being a group itself. This disjoint partitioning takes the form

2 $$FG_{i}H=\left\{F_{1}G_{i}H_{1},F_{1}G_{i}H_{2},...,F_{2}G_{i}H_{1},...\right\}$$

where F and H are subgroups of G . A given choice of subgroups is denoted by *R*. Each element of a double coset exhibits a degeneracy λ_*R*_, meaning that it appears λ_*R*_ times within the set. All elements within a double coset have the same degeneracy. Furthermore, for an Abelian group, this degeneracy coincides with the number of common elements between the two sets

$$\lambda_{R}=\left|F\cap H\right|$$

As such, for a choice *R*, all double cosets have the same degeneracy. Different choices of the central symmetry element *G*_*i*_ of a given PG in eq 2 may result in either distinct (no elements in common) or equivalent (all elements in common) sets. In order to systematically characterize the distinct sets, one arbitrary element *R*_*k*_ is selected from within each distinct double coset. These elements *R*_*k*_ are denoted as double-coset representatives (DCR) for a specific choice of subgroups F and H , with *n*_DCR_^*R*^ the number of distinct cosets. The selected DCRs can then be used to rewrite eq 1, thereby replacing *Ĝ*_*i*_ by *F̂*_*i*_ *R̂*_*k*_ *Ĥ*_*j*_. Furthermore, the redundant sum over all group elements ∑_*i*_^*n*_*G*_^ in eq 1 can be decomposed into the sum over elements of the subgroups ∑_*i*_^*n*_*F*_^ and ∑_*j*_^*n*_*H*_^, as well as the DCRs ∑_*k*_^*n*_DCR_^*R*^^. The resulting decomposed expression for the projection, i.e., the DCD, is

$${\mathrm{P̂}}^{\alpha }\chi_{\mu ,A}=\frac{1}{n_{G}\lambda_{R}}\overset{n_{F}}{\underset{i}{\sum }}\overset{n_{H}}{\underset{j}{\sum }}\overset{n_{\mathrm{DCR}}^{R}}{\underset{k}{\sum }}\gamma_{F_{i}}^{\alpha *}\gamma_{R_{k}}^{\alpha *}\gamma_{H_{j}}^{\alpha *}{\mathrm{F̂}}_{i}{\mathrm{R̂}}_{k}{Ĥ}_{j}\;\chi_{\mu ,A}$$ 3

The appropriate choice of the left and right subgroups is the key to achieving the desired factorization, as it allows the elimination of the corresponding sums over elements of the subgroups F and H , i.e., the sums over *i* and *j* in eq 3. It is stressed, that the remaining sum over *k* has fewer elements than the original sum in eq 1.

Next, it will be discussed how to arrive at a suitable choice for F and H , thereby introducing the concept of stabilizers. Let us first consider the action of the symmetry operation *Ĝ*_*i*_. By definition, its action on a function *f* of the 3D space vector **r** is

$${Ĝ}_{i}f\left(r\right)=f\left({Ĝ}_{i}^{-1}r\right)$$

with *G*_*i*_^–1^ as the inverse element of *G*_*i*_ acting on **r**. Hence, the action of *Ĝ*_*i*_ on an AO leads to a linear combination of functions from the same set

$${Ĝ}_{i}\;\chi_{\mu ,A}=\underset{\mu^{\prime }}{\sum }C_{\mu \mu \prime }^{A}\left(G_{i}\right)\chi_{\mu \prime ,G_{i}\left(A\right)}$$ 4

where *G*_*i*_(*A*) is the center into which *A* transforms after application of the symmetry operation *Ĝ*_*i*_. The coefficient matrices *C*_*μμ*′_^*A*^(*G*_*i*_) depend on the angular momenta of the basis functions and differ depending on whether Cartesian or spherical basis sets are used, respectively. In the latter case, for example, the coefficient matrices coincide with the Wigner-D matrices.^40^ In the general case, as seen in eq 4, the symmetry operation moves the original center of the AO function *A* to a *different* symmetry-equivalent center *G*_*i*_(*A*). *Some* symmetry operations, however, leave specific atomic centers invariant. More concretely, the set of all symmetry elements *U*_*i*_ of the full group G that leave a given atomic center *A* invariant form a subgroup U , which is referred to as the stabilizer of *A*. Thus, the choice of subgroups F and H for the DCD in eq 3 can be based on the stabilizers of the respective centers in order to precalculate the factorization mentioned above.

Having established the guiding principles underlying the DCD of the projection operator in eq 3, the elimination of redundant contributions in the integral calculation over SAOs is addressed in the following paragraphs. Note that the AO to SAO transformation is thereby performed implicitly.

A one-electron integral over SAOs ⟨*X*_μ,*A*_^α^|*Ô*|*X*_ν, *B*_^β^⟩ can be rewritten using the DCD in eq 3 as

5 $$\left\langle X_{\mu ,A}^{\alpha }\right|Ô\left|X_{\nu ,B}^{\beta }\right\rangle =\frac{1}{\lambda_{R}}\sum_{\gamma }I_{\alpha \gamma \beta }\sum_{\mu^{\prime }}\Lambda_{\mu \mu \prime }^{\alpha A*}\left(E\right)\sum_{j}^{n_{\mathrm{DCR}}^{R}}\sum_{\nu^{\prime }}\Lambda_{\nu \nu \prime }^{\beta B}\left(R_{j}\right)\left\langle \chi_{\mu \prime ,A}\right|{Ô}^{\gamma }\left|\chi_{\nu \prime ,R_{j}\left(B\right)}\right\rangle$$

where *E* is the identity symmetry operation. Note that in the above expression, the subgroups F and H of the double coset have been chosen as the stabilizers of *A* and *B*, respectively. The matrices Λ_*μμ*′_^*αA*^(*G*_*i*_) contain the corresponding sums over the elements of the subgroups

6 $$\Lambda_{\mu \mu \prime }^{\alpha A}\left(G_{i}\right)=\frac{1}{n_{G}}\sum_{k}^{n_{U}}\gamma_{G_{i}U_{k}}^{\alpha *}C_{\mu \mu \prime }^{A}\left(G_{i}U_{k}\right)$$

Note that in eq 6, the sum over *k* involves the elements *U*_*k*_ of the subgroup U , i.e., the stabilizer of *A*. The operator *Ô* ^γ^ in eq 5 is the result of a symmetry projection ${\mathrm{P̂}}^{\gamma }Ô$ of the original operator onto IRREP Γ^γ^. Thus, the sum over γ in eq 5 runs over all nonvanishing projections. Lastly, the prefactor *I*_*αγβ*_ acts as a selection rule: it equals *n*_*G*_ if the direct product Γ^α^ ⊗ Γ^γ*^ ⊗ Γ^β*^ corresponds to the totally symmetric IRREP Γ^1^, and is zero otherwise.

As mentioned earlier, choosing the left and right subgroups of the double coset as the stabilizers of *A* and *B* results in precalculating the final factorization of the redundant terms of the projection in advance. It can hence be recognized that the sum over the DCRs *R*_*j*_ only affects the ket of the integral, transforming the center of the respective AO function from *B* to *R*_*j*_(*B*). Through this restricted sum, the minimum number of nonredundant [*A*, *R*_*j*_ (*B*)] pairs needed for the calculation of the one-electron integral are generated.

Similarly to the one-electron case, the two-electron integrals over SAOs expressed in Mulliken notation, can be rewritten as

7 $$\begin{array}{l} \left(X_{\mu ,A}^{\alpha }X_{\nu ,B}^{\beta }|X_{\rho ,C}^{\gamma }X_{\sigma ,D}^{\delta }\right)=\frac{I_{\alpha \beta \gamma \delta }}{\lambda_{T}}\sum_{\mu^{\prime }\nu^{\prime }\rho^{\prime }\sigma^{\prime }}\Lambda_{\mu \mu \prime }^{\alpha A*}\left(E\right)\sum_{j}^{n_{\mathrm{DCR}}^{R}}\Lambda_{\nu \nu^{\prime }}^{\beta B}\left(R_{j}\right)\sum_{l}^{n_{\mathrm{DCR}}^{T}}\Lambda_{\rho \rho \prime }^{\gamma C*}\left(T_{l}\right)\sum_{k}^{n_{\mathrm{DCR}}^{S}}\Lambda_{\sigma \sigma^{\prime }}^{\delta D}\left(T_{l}S_{k}\right)\cdot \\ \left(\chi_{\mu \prime ,A}\chi_{\nu \prime ,R_{j}\left(B\right)}|\chi_{\rho \prime ,T_{l}\left(C\right)}\chi_{\sigma \prime ,T_{l}S_{k}\left(D\right)}\right) \end{array}$$

Here, *R*_*j*_ are the DCRs of the double cosets with the stabilizers of *A* and *B*, *S*_*k*_ are the DCRs of the double cosets with stabilizers of *C* and *D. T*_*l*_, on the other hand, are the DCRs of the double cosets with stabilizers of the center pairs [*A*, *R*_*j*_(*B*)] and [*C*, *S*_*k*_(*D*)]. The prefactor *I*_*αβγδ*_ acts as a selection rule associated with the direct product Γ^α^ ⊗ Γ^β*^ ⊗ Γ^γ^ ⊗ Γ^δ*^. Again, the nonredundant combinations of centers *A*, *B*, *C*, and *D* necessary for the calculation of the two-electron integrals are generated by the DCRs through the DCD.

A detailed derivation of the expressions in eqs 5 and 7 can be found in ref 22.

The main difference between the simplification from the general case to real or complex Abelian PGs, apart from the presence of complex-valued characters, lies in eq 4. In the case of real Abelian PGs, the coefficient matrix *C*_*μ μ*′_^*A*^(*G*_*i*_) always has one single nonvanishing element and can be simplified to a parity prefactor ± 1.^2,39^ Hence, the expressions for the one- and two- electron integrals simplify to

8 $$\left\langle X_{\mu ,A}^{\alpha }\right|Ô\left|X_{\nu ,B}^{\beta }\right\rangle =\frac{1}{\lambda_{R}}\sum_{\gamma }I_{\alpha \gamma \beta }\Lambda_{\mu \mu }^{\alpha A}\left(E\right)\sum_{j}^{n_{\mathrm{DCR}}^{R}}\Lambda_{\nu \nu }^{\beta B}\left(R_{j}\right)\left\langle \chi_{\mu ,A}\right|{Ô}^{\gamma }\left|\chi_{\nu ,R_{j}\left(B\right)}\right\rangle$$

and

9 $$\begin{array}{l} \left(X_{\mu ,A}^{\alpha }X_{\nu ,B}^{\beta }|X_{\rho ,C}^{\gamma }X_{\sigma ,D}^{\delta }\right)=\frac{I_{\alpha \beta \gamma \delta }}{\lambda_{T}}\Lambda_{\mu \mu }^{\alpha A}\left(E\right)\sum_{j}^{n_{\mathrm{DCR}}^{R}}\Lambda_{\nu \nu }^{\beta B}\left(R_{j}\right)\sum_{l}^{n_{\mathrm{DCR}}^{T}}\Lambda_{\rho \rho }^{\gamma C}\left(T_{l}\right)\sum_{k}^{n_{\mathrm{DCR}}^{S}}\Lambda_{\sigma \sigma }^{\delta D}\left(T_{l}S_{k}\right)\cdot \\ \left(\chi_{\mu ,A}\chi_{\nu ,R_{j}\left(B\right)}|\chi_{\rho ,T_{l}\left(C\right)}\chi_{\sigma ,T_{l}S_{k}\left(D\right)}\right) \end{array}$$

in the case of real Abelian PGs, respectively. In the case of complex Abelian PGs, however, the coefficient matrices possess more than one nonvanishing element. Specifically, the matrices of proper and improper rotations around the principal rotation axis which are present in these groups create a linear combination of AOs with the same total-angular momentum quantum number *l* for a given Gaussian exponent. The number of contributing functions in this linear combination is (*l* + 1)­(*l* + 2)/2 for a Cartesian basis and 2*l* + 1 for a spherical basis. In the latter case, this sum is further limited to a maximum of two contributions from AOs with the same absolute magnetic quantum number |*m*_*l*_| associated with angular momentum *l*, since only rotations over the principal rotation axis exist in complex Abelian PGs. As such, the sums over AOs in eqs 5 and 7 that arise from eq 4 do not simplify to one single contribution. Yet, they run over a limited number of AOs that can be easily predetermined.^41^

### B Complex Abelian Point Groups in Second Quantization

In this section, the symmetry properties of the targeted wave function in the case of complex Abelian PGs are examined within the second-quantization formalism. Note that, in contrast to real Abelian PGs, proper care must be taken to account for the complex conjugation of the matrix representations of the IRREPs. The discussion addresses the treatment of HF, CC, and EOM-CC approximations, as well as the evaluation of properties via the construction of density matrices.

In second quantization, a particle creation operator with respect to the true vacuum, i.e., *a*_*p*_^†^ can be associated with the Γ_*p*_ IRREP of the symmetry-adapted molecular orbital and an annihilator *a*_*p*_ with the IRREP corresponding to the complex conjugate matrices Γ_p_^*^. The IRREP of the Fermi vacuum,

$$\left|\Phi_{0}\right\rangle =\prod_{i}^{\mathrm{occ}}a_{i}^{†}\left|\mathrm{vac}\right\rangle$$

which in this discussion corresponds to the HF state, is determined by the direct product of the IRREPs of the occupied (*i*, *j*, *k*,...) orbitals

$$\Gamma_{\Phi_{0}}=\Gamma_{i}⊗\Gamma_{j}⊗...$$

In practical terms, one can select the IRREP of the HF state by choosing the number of occupied orbitals from each IRREP.

For an arbitrary operator expressed in second quantization

$$Ô=\sum_{\mathrm{pq}...\mathrm{rs}...}o_{\mathrm{rs}...}^{\mathrm{pq}...}a_{p}^{†}a_{q}^{†}...a_{s}a_{r}$$

each amplitude *o*_*rs*..._^*pq*···^ is associated with IRREP

$$\Gamma_{p}⊗\Gamma_{q}⊗...⊗{\left(\Gamma_{r}\right)}^{*}⊗{\left(\Gamma_{s}\right)}^{*}⊗...=\Gamma_{o_{\mathrm{rs}...}^{\mathrm{pq}...}}$$

If this operator is symmetry-adapted and transforms as Γ_*Ô*_, all amplitudes for which

$$\Gamma_{o_{\mathrm{rs}...}^{\mathrm{pq}...}}\neq \Gamma_{Ô.}$$

are zero and eq II B thus acts as a selection rule.

It is noted that the electronic Hamiltonian *Ĥ* and all its individual contributions transform as the totally symmetric IRREP Γ_*Ĥ*_ = Γ^1^. Furthermore, the operators used to describe electron correlation effects for the ground state by working on the HF reference are themselves totally symmetric. This follows from the fact that the correlated wave function has the same IRREP as the Fermi vacuum Γ_Φ_0__ = Γ_Ψ_corr__. One such example is the cluster operator T̂ in CC theory,^42^ i. e.,

$$\left|\Psi_{\mathrm{corr}}\right\rangle =e^{\mathrm{T̂}}\left|\Phi_{0}\right\rangle$$

In the EOM-CC approach,^42^ an excited state is expressed via an excitation starting from a correlated reference wave function

$$\left|\Psi_{\mathrm{exc}}\right\rangle =\mathrm{R̂}\left|\Psi_{\mathrm{corr}}\right\rangle$$

The IRREP of *R̂* can be determined via the IRREP of the targeted excited state as

$$\Gamma_{\mathrm{R̂}}=\Gamma_{\Psi_{\mathrm{exc}}}⊗\Gamma_{\Psi_{\mathrm{corr}}}^{*}$$

The respective deexcitation operator *L̂* transforms accordingly as Γ_*L̂*_ = $\Gamma_{\mathrm{R̂}}^{*}$ .

Lastly, it is noted that density matrices **D** typically used for the calculation of properties are necessarily associated with Γ^1^ irrespective of the IRREP of the electronic wave function, as they arise from expectation-value expressions. Transition-density matrices **D** ^(*m*) → (*n*)^ between states (*m*) and (*n*), however arise from integrals of the form ⟨Ψ^(*n*)^|···|Ψ^(*m*)^⟩ and transform as the IRREPs of the transition Γ_tr_ = $\Gamma_{\Psi^{\left(n\right)}}^{*}$ ⊗ Γ_Ψ^(*m*)^_. In the EOM-CC approach Γ_tr_ can be determined from the left and right EOM vectors as

$$\Gamma_{\mathrm{tr}}=\Gamma_{{\mathrm{L̂}}^{\left(n\right)}}⊗\Gamma_{{\mathrm{R̂}}^{\left(m\right)}}$$

### C Handling of Symmetry-Adapted Tensors

A major computational advantage of symmetry exploitation is the reduction of cost that can be achieved by the selection rule in eq II B. To exploit it, we use the following approach to handle the amplitude tensors: The orbital indices *p*, *q*,... are sorted according to their IRREP. This leads to a block structure of the tensors, where the IRREP of each index within a block is constant. Nonvanishing blocks with index *t* are given a tag number *t*_num_ and vanishing blocks are ignored. Assuming an *n*-dimensional tensor *O*_*p*_1_, *p*_2_,...*p*_*n*__ the tag of each block is

$$t_{\mathrm{num}}=\sum_{i=1}^{n}(\Gamma_{p_{i}}{)}_{\mathrm{num}}n_{G}^{i-1}$$

Here (Γ^α^)_num_ = α – 1 is the enumeration tag of the IRREPs with the totally symmetric IRREP as the first element (Γ^1^)_num_ = 0. For given dimensions of the tensor *n* and the order of the group *n*_*G*_, the tag is unique and directly identifies the IRREP of each index in the block. More importantly, the blocks are ordered in ascending tag number. This sorting allows the utilization of a binary search algorithm with logarithmic scaling, when a specific block needs to be selected. Furthermore, it is noted that for *n* indices a total of *n*_*G*_^*n*^ IRREP combinations are generated. If these combinations are restricted such that the direct product of the IRREPs yields the required representation, as dictated by the vanishing-integral rule, *n*_*G*_^*n*–1^ combinations remain, since the choice of the final index is fixed by the preceding *n*–1 indices. Thus, by considering only the *n*_*G*_^*n*–1^ nonvanishing blocks, the memory requirement is reduced proportional to $O\left(n_{G}\right)$ , i.e., instead of storing *N* ^*n*^ elements for the tensor, each block has ${\left(\frac{N}{n_{G}}\right)}^{n}$ elements in the case of equally distributed indices, resulting in $n_{G}^{n-1}{\left(\frac{N}{n_{G}}\right)}^{n}=\frac{N^{n}}{n_{G}}$ nonzero elements. *N* is the number of basis functions, which roughly corresponds to the system size. Accordingly, as resorting the entries of the tensors, often also called transposition-steps, scales linearly with the elements of the tensor, transposing a tensor is $O\left(n_{G}\right)$ more efficient when exploiting the block structure.

An important operation, which is needed when implementing working equations, is the tensor contraction

$$O_{p_{1},p_{2},...,q_{1},q_{2},...}=\sum_{s_{1},s_{2},...}P_{p_{1},p_{2},...,s_{1},s_{2},...}Q_{s_{1},s_{2},...,q_{1},q_{2},...}$$

The indices *p*_*i*_ and *q*_*i*_ will be referred to as target indices and *s*_*i*_ as summation indices. Note that the ordering of indices within the tensors **O**, **P**, and **Q** is irrelevant for the symmetry handling, but may be important for the actual contraction algorithm, as some algorithms require a transposition step.^43^

Owing to the block structure of the tensors **O**, **P**, and **Q**, the contraction is carried out block by block

$$O_{p_{1},p_{2},...,q_{1},q_{2},...}^{t}=\sum_{\left\{u,v\right\}}^{\mathrm{allowedpairs}}\sum_{s_{1},s_{2},...}P_{p_{1},p_{2},...,s_{1},s_{2},...}^{u}Q_{s_{1},s_{2},...,q_{1},q_{2},...}^{v}$$

where *t*, *u*, and *v* are the respective block indices. The target indices *p*_*i*_ and *q*_*i*_ in the *u* and *v* blocks of tensors **P** and **Q**, respectively, have to match with the target block *t* of **O**. For this reason, the sum over *u* and *v* is restricted to these matching blocks. Accordingly, not all *u*, *v* combinations are allowed since the summation indices *s*_*i*_ have to match as well. The algorithm for this operation thus has the following steps:

1. Loop over the *t* blocks of **O**.
2. Preselect the contributing blocks *u* of **P** based on the IRREPs of *p*_*i*_.
3. Preselect the contributing blocks *v* of **Q** based on the IRREPs of *q*_*i*_.
4. Identify the allowed {*u*, *v*} pairs from the preselected lists of indices.
5. Loop over the allowed pairs {*u*, *v*} and perform the contraction operation to the individual **P** ^*u*^ and **Q** ^*v*^ blocks, adding their contribution to block **O** ^*t*^ block.

In this way, the contraction in block structure exhibits savings at the order of $O\left(n_{G}^{2}\right)$ resulting from the following considerations: with *n*_*p*_, *n*_*q*_, and *n*_*s*_ the number of *p*_*i*_, *q*_*i*_, and *s*_*i*_ indices, respectively, instead of *N* ^*n*_*p*_+*n*_*q*_+*n*_*s*_^ floating point operations needed for the contraction, ${\left(\frac{N}{n_{G}}\right)}^{n_{p}+n_{q}+n_{s}}$ operations are performed for each block combination in the case of equally distributed indices. In order to find the number of allowed pair combinations *n*_pair_ we note that there are in total *n*_*G*_^*n*_*p*_+*n*_*q*_+*n*_*s*_^ distinct IRREP combinations for all *p*_*i*_, *q*_*i*_, and *s*_*i*_ indices. But since the {*p*_*i*_, *s*_*i*_} and the {*s*_*i*_, *q*_*i*_} combinations have each been reduced by *n*_*G*_ from the selection rule in the block structures of **P** and **Q**, as discussed in the previous paragraph, the number of allowed pairs is *n*_pair_ = *n*_*G*_^*n*_*p*_+*n*_*q*_+*n*_*s*_–2^. As such the total floating point operations in block structure are

$$n_{\mathrm{pair}}{\left(\frac{N}{n_{G}}\right)}^{n_{p}+n_{q}+n_{s}}=\frac{N^{n_{p}+n_{q}+n_{s}}}{n_{G}^{2}}$$

This up to quadratic decrease in floating-point operations resulting from symmetry handling is accompanied by the additional preselection steps 2–4 of the algorithm described above. However, their computational cost is negligible.

In most matrix multiplication routines which are commonly used for tensor contractions, including those in BLAS,^44^ optimal performance is generally achieved only for sufficiently large matrices. The symmetry-induced block structure, however, replaces a single large contraction by multiple contractions over smaller tensors, which reduces the efficiency of the BLAS routines and introduces a computational overhead. For larger systems, this effect is partially mitigated as the individual symmetry blocks themselves grow in size, thereby improving the efficiency of the underlying matrix multiplications. In addition, a more even distribution of tensor indices among the IRREPs enhances the effective reduction in computational cost.

In principle the individual symmetry blocks themselves can be handled independently of each other offering an opportunity for parallelization. However, such a parallel handling of the blocks directly competes with the highly optimized parallelization present within the underlying multiplication routines. In our experience, keeping the parallelization within the matrix multiplication step instead of the block structure is thus preferred. At the same time, the potential gains due to parallelization do not differ between the real and the complex Abelian PGs.

Preliminary calculations indicate that, for typical quantum-chemical applications up to the CCSDT truncation level, substantial computational savings are obtained for *n*_*G*_ ≤ 8 when exploiting complex Abelian PGs.

## III Implementation

The exploitation of complex Abelian PGs of order *n*_*G*_ ≤ 8 in the context of finite-magnetic field calculations has been implemented in the cfour^23,24^ and qcumbre^30,31^ program packages. The implemented PGs are *C*_*n*_, with *n* = 3–8, *C*_*nh*_ with *n* = 3, 4, and *S*_*n*_ with *n* = 4, 6, 8. In the present implementation, we restrict the symmetry to *n*_*G*_ ≤ 8, as the underlying program infrastructure for real Abelian point groups on which our approach builds is currently implemented only up to this value. In principle, this limitation is not fundamental and could be lifted. Potential advantages may arise for linear molecules (or atoms) oriented parallel to the magnetic field, which transform according to *C*_∞_ or *C*_∞*h*_. In such cases, the relevant consideration is the angular momentum quantum number *m*_*l*_ of the orbitals that defines their irreducible representations. However, for typical applications, the highest occupied angular momentum is usually *m*_*l*_ = ± 3 (ϕ orbitals), for which a PG with a principal rotation axis of cardinality 6 already suffices to distinguish the corresponding IRREPs.^45^ Higher orders would introduce additional representations that remain largely unoccupied. Consequently, extending to *n*_*G*_ > 8 is not expected to provide significant computational or interpretational advantages.

The DCD as described in Section II 2.1 is utilized in the mint module^27^ of cfour for the calculation of integrals over London orbitals using the McMurchie-Davidson algorithm.^15,46^ The implementation is based on a pre-existing implementation by Gauss for the case of real Abelian PGs. We note that the use of complex Abelian PGs was also extended for the use with the Cholesky decomposition of the two-electron integrals over London orbitals.^33,47,48^ The implicitly generated SAOs by the DCD are employed in ff-SCF calculations within cfour.

The block structure as described in Section II 2.3 has been implemented in the back-end of qcumbre.^30^ As such, all currently available post-HF approaches within the program are able to exploit both real and complex Abelian PGs. Future implementations within qcumbre hence only need to explicitly consider the IRREP of the involved tensors and operators in order to function with the presented block structure. Further details on the implementations within cfour and qcumbre can be found in ref 22.

The validity of the implementation was verified by comparing energies and molecular properties obtained at both the SCF and correlated levels of theory. Results from calculations employing complex and real Abelian PGs were benchmarked against corresponding calculations performed without symmetry exploitation.

## IV Computational Details

To demonstrate the computational speedup achieved by exploiting the implemented complex Abelian PGs, quantum-chemical calculations at the ff-CCSD level of theory using London orbitals were performed on four molecular systems. A convergence criterion of 10^–8^ a.u. was used for the underlying SCF calculation for the norm of the error vector of the SCF-DIIS procedure and a convergence criterion of 10^–7^ was employed for the solution to the CCSD equations for the norm of the difference in cluster amplitudes between two consecutive iterations. The systems considered are small hydrocarbons in the presence of a magnetic field in an orientation that exhibits a complex Abelian PG symmetry. Specifically, the molecules studied are (1) CH_4_ in a standard tetrahedral configuration (C_3_), (2) CH_4_ in a planar configuration (C_4h_), (3) CH_3_–CH_3_ in a staggered configuration (S_6_), and (4) CH_2_=C=CH_2_ (S_4_). The molecules were chosen mainly for their symmetry properties though they might be of interest for atmospheres of DQ white dwarf stars where CH and C_2_ bands are have been recorded.^17,34,35,49–51^ Note that while planar methane, system (2), for example, does not correspond to an equilibrium structure of the molecule, methane may drastically deform in the presence of a magnetic field and acquire a planar fan-like geometry for strong magnetic fields.^21^ The employed orientation of the magnetic field in the aforementioned systems are depicted in Table [Table tblII]. The magnetic field strength was *B* = 0.1 *B*_0_ for systems (1–3) and *B* = 0.001 *B*_0_ for system (4).^52^

**2 tblII:**
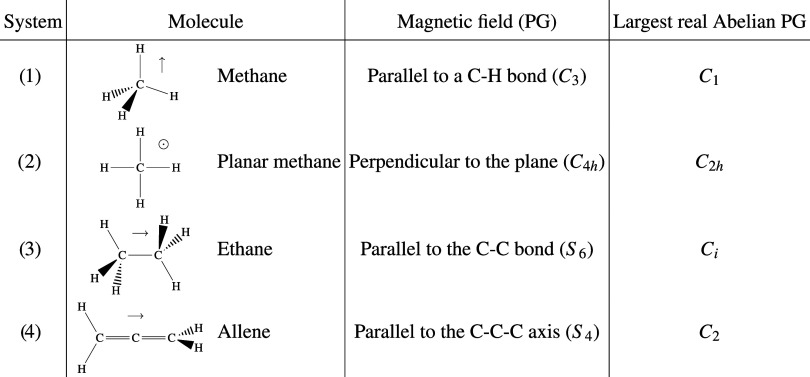
Molecular Systems Used to Assess Computational Cost Reduction via Complex Abelian Point-Group Symmetry

The calculation of integrals and the solution of the HF equations were performed using the cfour program package.^23,24,27^ The transformation of integrals from the (S)­AO to the molecular-orbital (MO) basis as well as the CCSD calculation were performed with the qcumbre program.^30^ The correlation-consistent cc-pV*X*Z basis sets, with *X* = D, T, Q, were employed.^53^ Calculations were performed using the full complex PG and the largest real Abelian subgroup of each system, as listed in the same table. The resulting computational times are compared with those obtained from calculations without symmetry exploitation, corresponding to *C*_1_ as computational PG. To ensure a meaningful comparison of computational timings, all calculations for a given system were executed on a single node using a single thread, with no concurrent jobs running. Specifically, calculations for system (1) and (2) were performed on an Intel­(R) Xeon­(R) CPU E5-2643 v43.40 GHz, CPU 12 core, memory 1,5 TB, disk 6 TB architecture, while systems (3) and (4) were performed on an Intel­(R) Xeon­(R) CPU E5-2699 v42.20 GHz, CPU 44 core, memory 756 GB, disk 6 TB architecture.

## V Results

The speedup observed for calculations on systems (1–4) upon exploiting molecular symmetry is reported in Table [Table tblIII]. The individual steps of a CCSD calculation were analyzed separately. These steps include: (a) the evaluation of integrals over London orbitals, which scales as $O\left(N^{4}\right)$ , (b) the solution of the HF problem using an SCF algorithm scaling as $O\left(N^{4}\right)$ as well, (c) the transformation of integrals from the (S)­AO basis to the molecular orbital (MO) basis, scaling as $O\left(N^{5}\right)$ , and (d) the solution of the CCSD equations, which scales as $O\left(N^{6}\right)$ . It is worth noting that, at the algorithmic level, only the integral calculation using the DCD approach differs between the complex and real Abelian PG implementations. The symmetry handling of all other steps is based on a unified implementation for both cases. To quantify the speedup, execution times from calculations performed in *C*_1_ are compared to those obtained when molecular symmetry is exploited, giving the ratio

$$r=\frac{t\left(C_{1}\right)}{t\left(\mathrm{Comp.PG}\right)}$$

This ratio can be found in the central columns of Table [Table tblIII]. To further evaluate the implementation and relate it to the theoretically derived cost reduction discussed in Section II 2.3, the ratio is examined in terms of powers of the point-group order *n*_*G*_. To assess whether the expected up to quadratic behavior is observed numerically, the exponent log_*n*_*G*__(*r*) which is related to the ratio by

$$r=n_{G}^{\log_{n_{G}}\left(r\right)}$$

is reported in the columns next to the ratios in Table [Table tblIII]. Lastly, in the rightmost columns the number of iterations needed to achieve convergence for the SCF and CCSD steps are presented. For the integral evaluation, the speedup due to the use of the DCD is highly system specific and does not depend on *n*_*G*_ directly, but instead on the number of symmetry equivalent centers, as discussed on the topic of stabilizers in Section II 2.1. The speedup of all other steps stems mainly from the block structure of the tensors involved and the contractions between them. Note that for very short time steps, the timing resolution of the qcumbre calculations did not permit the evaluation of the ratio.

**3 tblIII:** Reduction in the Time Needed to Complete a Calculation at the CCSD Level for Systems (1–4)

basis set (# AOs)	comp. PG (*n*_*G*_)	ratio *r* (*C*_1_/comp. PG)					log_*n*_*G*__(*r*)					# of iterations	
		integrals	SCF	AO to MO	CCSD	total	integrals	SCF	AO to MO	CCSD	total	SCF	CCSD
(1) tetrahedral methane CH_4_													
cc-pVDZ (34)	*C*_3_ (3)	2.2	3.0	-[Table-fn tIIIfn1]	2.0	1.5	0.72	1.00	-[Table-fn tIIIfn1]	0.63	0.37	14	10
cc-pVTZ (86)	*C*_3_ (3)	2.4	7.1	5.6	4.3	3.8	0.80	1.78	1.57	1.33	1.22	14	10
cc-pVQZ (175)	*C*_3_ (3)	2.6	6.6	5.4	3.9	3.9	0.87	1.72	1.54	1.24	1.24	14	10
(2) planar methane CH_4_													
cc-pVDZ (34)	*C*_2*h*_ (4)	2.1	4.0	-[Table-fn tIIIfn1]	2.0	1.5	0.54	1.00	-[Table-fn tIIIfn1]	0.50	0.29	13	11
	*C*_4*h*_ (8)	2.2	8.0	-[Table-fn tIIIfn1]	1.0	1.2	0.38	1.00	-[Table-fn tIIIfn1]	0.00	0.09	13	11
cc-pVTZ (86)	*C*_2*h*_ (4)	2.4	9.2	7.3	6.3	4.7	0.63	1.60	1.43	1.33	1.12	14	11
	*C*_4*h*_ (8)	2.8	34.1	29.0	5.7	5.4	0.50	1.70	1.62	0.84	0.81	14	11
cc-pVQZ (175)	*C*_2*h*_ (4)	2.5	9.7	7.2	6.0	5.0	0.66	1.64	1.42	1.29	1.16	14	11
	*C*_4*h*_ (8)	4.1	34.0	25.6	7.9	8.9	0.68	1.70	1.56	0.99	1.05	15	11
(3) staggered ethane CH_3_–CH_3_													
cc-pVDZ (58)	*C*_*i*_ (2)	2.0	3.1	2.5	3.3	2.6	1.00	1.63	1.32	1.72	1.38	13	11
	*S*_6_ (6)	2.6	17.0	-[Table-fn tIIIfn1]	6.6	4.5	0.53	1.58	-[Table-fn tIIIfn1]	1.05	0.84	15	11
cc-pVTZ (144)	*C*_*i*_ (2)	2.2	3.1	2.6	2.8	2.6	1.14	1.63	1.38	1.49	1.38	14	11
	*S*_6_ (6)	2.3	21.2	10.8	10.9	7.6	0.46	1.70	1.33	1.33	1.13	16	11
cc-pVQZ (290)	*C*_*i*_ (2)	2.9	3.3	3.7	2.7	3.0	1.54	1.72	1.89	1.43	1.58	14	11
	*S*_6_ (6)	3.7	23.3	16.0	8.9	8.7	0.73	1.76	1.55	1.22	1.21	16	11
(4) allene CH_2_=C = CH_2_													
cc-pVDZ (62)	*C*_2_ (2)	1.7	3.0	2.3	3.6	3.0	0.77	1.58	1.20	1.85	1.58	17	13
	*S*_4_ (4)	3.0	11.0	7.0	7.1	5.8	0.79	1.73	1.40	1.41	1.27	18	13
cc-pVTZ (146)	*C*_2_ (2)	1.7	3.1	2.8	2.9	2.7	0.77	1.63	1.49	1.54	1.43	17	13
	*S*_4_ (4)	3.5	11.1	6.5	7.8	6.7	0.90	1.74	1.35	1.48	1.37	18	13
cc-pVQZ (290)	*C*_2_ (2)	1.8	3.1	3.4	2.7	2.7	0.85	1.63	1.77	1.43	1.43	17	13
	*S*_4_ (4)	4.3	11.6	8.7	6.6	6.5	1.05	1.77	1.56	1.36	1.35	18	13

a For very short time steps, the timing resolution of the qcumbre calculations did not permit the evaluation of the ratio.

It is observed that the cost reduction is greater for larger systems. This expected trend is explained by the fact that as the system size increases, a more balanced distribution of matrix indices among IRREPs enhances the computational efficiency achieved by excluding vanishing blocks. Moreover, the employed BLAS^44^ routines in this work perform better for larger matrices.

The integral calculation shows the least speedup compared to the other computational steps. For the existing implementation employing real Abelian PGs,^27^ the largest speedup for the integral evaluation is observed for system (3) with *n*_*G*_^1.54^ employing the *C*_*i*_ PG. The lowest speedup for this step is *n*_*G*_^0.54^. On average, for the integral evaluation, a value of *n*_*G*_^0.88^ is obtained. The newly implemented complex Abelian PGs underperform in comparison. This reduced efficiency of complex Abelian PGs arises from the fact that for the real Abelian PG case, the final DCD expressions involve simple parity factors, cf. eqs 8 and 9. For complex Abelian PG, on the other hand, the sums over basis functions arising from eq 4 cannot be simplified further, cf. eqs 5 and 7. However, it should be noted that the PGs *S*_4_ and *C*_4*h*_ are special cases where eq 4 has only one single contribution as well, and hence a better performance compared to other complex groups is expected. Excluding these cases, a maximum value *n*_*G*_^0.87^ is observed for system (1) computed within *C*_3_. The poorest performance is observed for system (2) calculated within *C*_4*h*_ using the cc-pVDZ basis set with *n*_*G*_^0.38^. The average of the exponent amounts to *n*_*G*_^0.70^ for the complex Abelian PGs for the integral evaluation step.

At the SCF level of theory, the complex Abelian PGs slightly outperform the real ones. The average of the exponents is *n*_*G*_^1.56^ for real PGs and *n*_*G*_^1.60^ for complex PGs, with maximum values of *n*_*G*_^1.72^ and *n*_*G*_^1.78^, respectively. The least reduction is in both cases *n*_*G*_^1.00^. This mildly better performance of the complex Abelian groups is observed even for the cases where up to two more iterations are needed in the case of complex Abelian PGs, as seen in Table [Table tblIII].

Both the integral transformation and the CCSD iterations are performed within qcumbre. For the integral transformation, an average speedup of approximately *n*_*G*_^1.49^ is observed for real and *n*_*G*_^1.50^ for complex PGs. For the CCSD iterations, more pronounced differences are found. The average speedup amounts to *n*_*G*_^1.07^ for complex PGs and *n*_*G*_^1.40^ for real PGs. Although this difference appears substantial, it is important to note that the calculation is much more efficient when using the complex PG even if the exponent of the ratio in the base of *n*_*G*_ is smaller. To explain this difference and the deviation from the quadratic speedup, we note that in the implementation used, tensor contractions are performed using BLAS matrix multiplication routines.^44^ As discussed in Section II 2.3, these multiplication routines exhibit optimal performance for larger matrices and, as such, are less efficient for larger groups in a given system. Furthermore, we note that, in the examples discussed here, the occupied orbitals are distributed rather inhomogeneously among the IRREPs for the complex Abelian PGs, which leads to a reduced efficiency as well. Because both real and complex PGs are treated within the same implementation and the number of CCSD iterations does not differ, the observed difference is attributed to the lower order of the real PGs rather than to an intrinsic disadvantage of complex Abelian PGs. For comparable group orders *n*_*G*_, or for larger systems, the aforementioned differences should diminish.

The overall speedup observed reflects the reduction in computational effort associated with solving the CCSD amplitude equations, which constitute the computationally most demanding part of the procedure. The average scaling exponents obtained are *n*_*G*_^1.00^ and *n*_*G*_^1.26^ for complex and real PGs, respectively. While the ideal theoretical scaling of *n*_*G*_^2^ is not fully reached, the observed speedup remains substantial. Deviations from the asymptotic limit are largely due to the small sizes of individual tensor blocks, which reduce the efficiency of matrix multiplication routines. Overall, the measured speedups demonstrate that symmetry exploitation provides significant and practically relevant performance gains.

It is also worth mentioning that, in an absolute sense, the complex Abelian PGs always outperform the use of the largest Abelian subgroup as seen by the calculated ratios. For example, for allene using a cc-pVQZ basis, a full CCSD calculation without symmetry exploitation lasted about 7 h and 11 min. Exploiting *C*_2_ reduced this to 2 h and 41 min, and fully exploiting *S*_4_ to 1 h and 6 min. Further details may be found in ref 22. The reported maximum RSS (resident set size, i.e., physical random access memory) values for the same system are 807.4 GB for *C*_1_, 269.4 GB for *C*_2_, and 140.5 GB for *S*_4_ and show the memory savings discussed in Section II 2.3. Lastly, while the present examples are limited to small molecules, calculations illustrating the symmetry reductions have also been performed for larger systems at the CCSD level, such as benzene in a magnetic field perpendicular to the molecular plane: Using a cc-pVTZ basis, a CCSD calculation without symmetry lasted 9 h, 55 min, 1 h and 35 min when using *C*_2*h*_, 58 min, 1 h for *C*_3*h*_, and 1 h and 11 min using *C*_6_, respectively. The corresponding maximum RSS values are 575.6 GB, 99.1 GB, 65.7 GB, and 53.9 GB.

Finally, we note that the exploitation of symmetry not only reduces computational cost, but it further facilitates the analysis of orbitals and excited states thereby acting as an interpretational tool, and enables the selective targeting of states belonging to specific IRREPs. Beyond ff-CCSD, we have performed calculations exploiting complex PGs for excited states using the EOM-CC ansatz.^17,22,34,35,48^ In these studies, the use of the aforementioned groups and identification of the IRREPs of the electronic states involved played a crucial role. We mention for example the study of B­(OH)_3_ in ref 17, a molecule that exhibits a complex PG even in the absence of a magnetic field. Here, the HOMO–LUMO transition belongs to IRREPs with complex characters and is *accidentally* doubly degenerate in the field-free case. As such, targeting these states at the EOM-CCSD level is impossible when using a real implementation. Using the complex Abelian PGs with our complex code facilitated their targeting and analysis.

## VI Conclusion

In this work, an implementation of complex Abelian PGs for quantum-chemical calculations in the context of finite-magnetic-field approaches was presented. The theoretical aspects presented cover the totality of an electronic-structure treatment, from the calculation of integrals over atom-centered basis functions to considerations for SCF and post-HF methodologies.

The implemented approaches were applied to small hydrocarbons in the presence of a finite magnetic field. Calculations at the ff-CCSD level were carried out to illustrate the associated decrease in computational cost. It can be concluded that, for a quantum-chemical code employing complex algebra in the context of finite magnetic fields, the use of complex Abelian PGs consistently provides significant efficiency gains over real Abelian subgroups, owing to the larger number of symmetry elements typically encountered as compared to the real Abelian subgroups. While the implementation presented here is limited to the case of finite-magnetic field approaches, similar gains are expected to be observed for other cases where complex wave functions occur.

## Data Availability

The data that support the findings of this study are available within the article.
